# Evidence for *STAT4* as a Common Autoimmune Gene: rs7574865 Is Associated with Colonic Crohn's Disease and Early Disease Onset

**DOI:** 10.1371/journal.pone.0010373

**Published:** 2010-04-29

**Authors:** Jürgen Glas, Julia Seiderer, Melinda Nagy, Christoph Fries, Florian Beigel, Maria Weidinger, Simone Pfennig, Wolfram Klein, Jörg T. Epplen, Peter Lohse, Matthias Folwaczny, Burkhard Göke, Thomas Ochsenkühn, Julia Diegelmann, Bertram Müller-Myhsok, Darina Roeske, Stephan Brand

**Affiliations:** 1 Department of Medicine II - Grosshadern, University of Munich, Munich, Germany; 2 Department of Preventive Dentistry and Periodontology, University of Munich, Munich, Germany; 3 Institute of Human Genetics, RWTH Aachen, Aachen, Germany; 4 Department of Human Genetics, Ruhr-University Bochum, Bochum, Germany; 5 Institute of Clinical Chemistry - Grosshadern, University of Munich, Munich, Germany; 6 Max-Planck-Institute of Psychiatry, Munich, Germany; New York University, United States of America

## Abstract

**Background:**

Recent studies demonstrated an association of *STAT4* variants with systemic lupus erythematosus (SLE) and rheumatoid arthritis (RA), indicating that multiple autoimmune diseases share common susceptibility genes. We therefore investigated the influence of *STAT4* variants on the susceptibility and phenotype of inflammatory bowel diseases (IBD) in a large patient and control cohort.

**Methodology/Principal Findings:**

Genomic DNA from 2704 individuals of Caucasian origin including 857 patients with Crohn's disease (CD), 464 patients with ulcerative colitis (UC), and 1383 healthy, unrelated controls was analyzed for seven SNPs in the *STAT4* gene (rs11889341, rs7574865, rs7568275, rs8179673, rs10181656, rs7582694, rs10174238). In addition, a detailed genotype-phenotype analysis was performed. Our analysis revealed an association of the *STAT4* SNP rs7574865 with overall decreased susceptibility to CD (p = 0.047, OR 0.86 [95% CI 0.74–0.99]). However, compared to CD patients carrying the wild type genotype, the *STAT4* SNP rs7574865 was significantly associated with early CD onset (p = 0.021) and colonic CD (p = 0.008; OR = 4.60, 95% CI 1.63–12.96). For two other *STAT4* variants, there was a trend towards protection against CD susceptibility (rs7568275, p = 0.058, OR 0.86 [95% CI 0.74–1.00]; rs10174238, p = 0.057, OR 0.86 [95% CI 0.75–1.00]). In contrast, we did not observe any association with UC susceptibility. Evidence for weak gene-gene interaction of *STAT4* with the *IL23R* SNP rs11209026 was lost after Bonferroni correction.

**Conclusions/Significance:**

Our results identified the *STAT4* SNP rs7574865 as a disease-modifying gene variant in colonic CD. However, in contrast to SLE and RA, the effect of rs7574865 on CD susceptibility is only weak.

## Introduction

The precise etiology of inflammatory bowel disease (IBD) is not completely understood but accumulating data on genetic risk factors, including genome-wide association studies, have significantly advanced our understanding of its pathogenesis. [1], [2] During the last decade, substantial progress has been made in identifying susceptibility genes for Crohn's disease (CD) involved in innate immunity (particularly genetic variants in the *NOD2/CARD15* region) [3]–[7] and autophagy (variants in the *ATG16L1* [8]–[11] and *IRGM* region) [12] as well as in the proinflammatory IL-23 [13], [14] and T-helper cell type 17 (Th17) pathway, [15], [16] highlighting the complex interaction between gut homeostasis, bacterial recognition and proinflammatory mucosal immune response in IBD. Moreover, it is now clear that adaptive immune responses involved in the IBD pathogenesis are more complex than the traditional dichotomous Th1/Th2 paradigm. Particularly the identification of the IL-23-Th17 pathway highlights the importance of T-cell differentiation in maintaining intestinal immune homeostasis in the pathogenesis of both CD and ulcerative colitis (UC). [16]–[18]

STAT4 (signal transducers and activators of transcription-4) represents a transcription factor transducing IL-12-, IL-23-, and type 1 interferon-mediated signals into Th1 and Th17 differentiation, monocyte activation, and interferon-gamma production. [19]–[23] The requirement for STAT4-dependent cytokine regulation has well been replicated for the pathogenesis of autoimmune encephalomyelitis, [24], [25] rheumatoid arthritis, [26], [27] and also IBD, [28]–[30] highlighting a critical role for STAT4 in autoimmune diseases. [23] Previous studies demonstrated constitutive STAT4 activation in intestinal T cells of CD patients [28] and an increased expression and activation of IL-12-induced STAT4 signaling in the mucosa of patients with UC. [29] Interestingly, STAT4 isoforms differentially regulate Th1 cytokine production and thereby the severity of IBD. [30] Using a transfer colitis model, it has been shown that particularly STAT4beta promotes colonic inflammation and tissue destruction which correlates with STAT4 isoform-dependent expression of TNF-α and GM-CSF *in vitro* and *in vivo*. [30] A recent *in vivo* study [31] demonstrated an impaired development of human Th1 cells in patients with deficient expression of STAT4.

Investigating the genetic background of STAT4 regulation, very recent studies suggested a significant association of genetic variants in the *STAT4* gene on chromosome 2q with systemic lupus erythematosus (SLE) and rheumatoid arthritis [23], [27], [32], [33] as well as Sjögren's disease (SD), [34] systemic sclerosis, [23], [35] psoriasis [36] and also type-1 diabetes, [37] thus indicating common genetic and molecular pathways in multiple autoimmune diseases. In patients with IBD, there are so far only limited data including a study on 700 Spanish IBD patients reporting a significant association of the *STAT4* variant rs7574865 with both susceptibility to CD and UC. [38] However, this association has not been replicated in larger patient cohorts of different ethnic origin and the phenotypic consequences are unknown so far. We therefore initiated a large genotype-phenotype analysis including 1321 Caucasian IBD patients (857 patients with CD, 464 patients with UC) and 1383 healthy, unrelated controls investigating genetic variants in *STAT4* as potential susceptibility genes in IBD and potential phenotypic consequences.

## Methods

### Study population and assessment of disease phenotype

The study population (n = 2704) consisted of 1321 Caucasian IBD patients including 857 patients with CD, 464 patients with UC, and 1383 healthy, unrelated controls. Written, informed consent was obtained from all patients prior to the study. The study was approved by the Ethics committee of the Ludwig-Maximilians-University Munich (Department of Medicine, Munich-Grosshadern) and adhered to the ethical principles for medical research involving human subjects of the Helsinki Declaration (http://www.wma.net/e/policy/b3.htm). For the diagnosis of CD or UC, established diagnostic guidelines including endoscopic, radiological, and histopathological criteria were used. [39] Patients with CD were assessed according to the Montreal classification [40] based on age at diagnosis (A), location (L), and behaviour (B) of disease. In patients with UC, anatomic location was also assessed in accordance to the Montreal classification, using the criteria ulcerative proctitis (E1), left-sided UC (distal UC; E2), and extensive UC (pancolitis; E3). Patients with indeterminate colitis were excluded from the study. Phenotypic characteristics included demographic data and clinical parameters (behaviour and anatomic location of IBD, disease-related complications, previous surgery or immunosuppressive therapy) which were recorded by investigation of patient charts and a detailed questionnaire including an interview at time of enrolment. All phenotypic data were collected blind to the results of the genotypic data. The demographic characteristics of the IBD study population are summarized in Table 1.

**Table 1:** Demographic characteristics of the IBD study population.

	Crohn's disease*N = 857*	Ulcerative colitis*n = 464*	Controls*n = 1383*
**Gender**			
Male (%)	45.3	47.9	62.6
Female (%)	54.7	52.5	37.4
**Age** (yrs)			
Mean ± SD	40.2±13.2	42.4±14.4	45.8±10.7
Range	11–81	7–86	18–71
**Body mass index**			
Mean ± SD	23.1±4.2	23.9±4.1	
Range	13–40	15–41	
**Age at diagnosis** (yrs)			
Mean ± SD	27.7±11.8	32.0±13.3	
Range	1–78	9–81	
**Disease duration** (yrs)			
Mean ± SD	11.9±8.6	10.5±7.7	
Range	0–44	1–40	
**Positive family history of IBD** (%)	16	16.1	

### DNA extraction and genotyping of the *STAT4* variants

From all study participants, blood samples were taken and genomic DNA was isolated from peripheral blood leukocytes using the DNA blood mini kit from Qiagen (Hilden, Germany) according to the manufacturer's guidelines. Seven *STAT4* SNPs (rs11889341, rs7574865, rs7568275, rs8179673, rs10181656, rs7582694, rs10174238) were genotyped by PCR and melting curve analysis using a pair of fluorescence resonance energy transfer (FRET) probes in a LightCycler® 480 Instrument (Roche Diagnostics, Mannheim, Germany). The SNP selection was based on a previous study demonstrating significant associations of these SNPs with SLE and rheumatoid arthritis. [27] The donor fluorescent molecule (fluorescein) at 3′-end of the sensor probe is excited at its specific fluorescence excitation wavelength (533 nm) and the energy is transferred to the acceptor fluorescent molecule at the 5′-end (LightCycler Red 610, 640 or 670) of the anchor probe. The specific fluorescence signal emitted by the acceptor molecule is detected by the optical unit of the LightCycler 480 Instrument. The sensor probe is exactly matching to one allele of each SNP, preferentially to the rarer allele, whereas in the case of the other allele there is a mismatch resulting in a lower melting temperature. The total volume of the PCR was 5 µl containing 25 ng of genomic DNA, 1× Light Cycler 480 Genotyping Master (Roche Diagnostics), 2.5 pmol of each primer and 0.75 pmol of each FRET probe (TIB MOLBIOL, Berlin, Germany). In the case of rs11889341, rs7574865, rs7568275 and rs8179673, the concentration of the forward primer, and in the case of rs7582694, the concentration of the reverse primer, were reduced to 1.25 pmol. For rs10181656, the concentration of the forward primer, and for rs10174238 the concentration of the reverse primer, were reduced to 0.5 pmol. The PCR comprised an initial denaturation step (95°C for 10 min) and 45 cycles (95°C for 10°C sec, 60 for 10 sec, 72°C for 15 sec). The melting curve analysis comprised an initial denaturation step (95°C for 1 min), a step rapidly lowering the temperature to 40°C and holding for 2 min, and a heating step slowly (1 acquisition/°C) increasing the temperature up to 95°C and continuously measuring the fluorescence intensity. The results of melting curve analysis have been confirmed by analyzing two patient samples for each possible genotype using sequence analysis. For sequencing, the total volume of the PCR was 100 µl containing 250 ng of genomic DNA, 1× PCR-buffer (Qiagen, Hilden, Germany), a final MgCl_2_ concentration of 1.5 mM, 0.2 mM of a dNTP-Mix (Sigma, Steinheim, Germany), 2.5 units of HotStar Plus Taq™ DNA polymerase (Qiagen) and 10 pmol of each primer (TIB MOLBIOL). The PCR comprised an initial denaturation step (95°C for 5 min), 35 cycles (denaturation at 94°C for 30 sec, primer annealing at 60°C for 30 sec, extension at 72°C for 30 sec) and a final extension step (72°C for 10 min). The PCR products were purified using the QIAquick PCR Purification Kit (Qiagen) and sequenced by a commercial sequencing company (Sequiserve, Vaterstetten, Germany). All sequences of primers and FRET probes and primer annealing temperatures used for genotyping and for sequence analysis are given in Tables 2 and 3. The genotype data for 10 IBD-associated *IL23R* SNPs (rs1004819, rs7517847, rs10489629, rs2201841, rs11465804, rs11209026 = p.Arg381Gln, rs1343151, rs10889677, rs11209032, rs1495965) were available from a previous study. [14]

**Table 2:** Primer sequences (F: forward primer, R: reverse primer), FRET probe sequences, and primer annealing temperatures used for *STAT4* genotyping.

Polymorphism	Primer sequences	FRET probe sequences
rs11889341	F: TCATTTTTTTCCACATGTCTACR: GAATGGAGTCCAGAAACAAG	TGAACATCTTATTCTTTTACCACTGC-FLLC610-CTGCTGGGCCAGCTCTGTCA
rs7574865	F: TTATGGAAAATTACATGAGTGTG GCAAATCTTTGTAAAAAGTCAA	GGTGACCAAAATGTTAATAGTGGT-FLLC640-ATCTTATTTCAGTGGAATTTCAGGGGAT
rs7568275	F: CCAACAATATTTTAGCCCTAGTCTAR: AGCCCAAAATCAATAACCAG	ACTTTATATGTTCAGTTAATATTACTTGGT-FLLC670-CATACTGACCCATGAGTGTTCAATTG
rs8179673	F: GCCATAGAAGTCTTTGAAGCTGAR: GTTTTTACCAGTTGGCAGCA	AAGTATATATTAAACAAAGGTCCATAACC-FLLC610-CCATACACATCATCATCATTTTAT *
rs10181656	F: AGTTTTCAAAGTCTAACACTGTGR: GCTGCCATGTCGAGAGTA	AACTCTTCTCACCCCTTGTACC-FLLC640-CTACCCTCCTTTGTAGCCTGGCTT
rs7582694	F: AACCTTCCAATGGTTTCTCAR: AAAGAACAAGCAAACATCCATAG **	GTTGCATACTCTATGTGTCATCCC-FLLC670-TCATGAATTTGGTGTGGGTAGAACAAT
rs10174238	F: GTTGAGAGAGGAGAATCTGTTGAACCR: ATGAACAAGACTGTCACTATCGAG	AAGGTAAACAATGATAACGTCTTTTT-FLLC610-TTTTGAGATAGAGTTTCAATCTGTCACCCA

Note: FL: Fluorescein, LC610: LightCycler-Red 610; LC640: LightCycler-Red 640. The polymorphic position within the sensor probe is underlined. A phosphate is linked to the 3′-end of the acceptor probe to prevent elongation by the DNA polymerase in the PCR.

*The underlined T bases within the rs8179673 anchor probe represent LNA (locked nucleic acid) bases in.

**The underlined C base within the rs7582694 reverse primer differs from the original sequence.

**Table 3:** Primer sequences used for the sequence analysis of *STAT4* variants.

Polymorphism	Primer sequences
rs11889341	CAAATACCTTCTACTCTATGGCT GAATGGAGTCCAGAAACAAG
rs7574865	AAAGAAGTTTGTAATTAAAAAGCTA GCAAATCTTTGTAAAAAGTCAA
rs7568275	AAACCAACCTCATTAAATTATAGCA GAAGACAAAAAGAAAATATGGTCAC
rs8179673	TTAGTTTTTCCCACGTTTAGTTCTC TCAAATCATATGGGGAGGAGTG
rs10181656	GTGGATAAAAATACAAAATAAAAGAC GGATTTCAACAGGATCACC
rs7582694	AACCTTCCAATGGTTTCTCA CAGTGGATATGGATTTTAGTACTCTG
rs10174238	GAGGTAGAGGTTACAGTGAGCCGA AAGCAATGTTTCACCTGTCCCT

### Statistical analyses

For data evaluation, we used the SPSS 13.0 software (SPSS Inc., Chicago, IL, U.S.A.) and R-2.4.1. (http://cran.r-project.org). Each genetic marker was tested for Hardy-Weinberg equilibrium in the control population. Fisher's exact test was used for comparison between categorical variables, while Student's t test was applied for quantitative variables. Single-marker allelic tests were performed with Pearson's χ^2^ test. All tests were two-tailed, considering p-values<0.05 as significant. Odds ratios were calculated for the minor allele at each SNP. Bonferroni correction was applied for multiple comparisons as indicated. LD was calculated using the library genetics implement in R (http://www.r-project.org). We also used R for interaction analysis using the allelic model. Haplotypes were calculated using a sliding-window approach varying the window size from 2 to 7 included SNPs and using the option “hap-logistic” as implemented in PLINK (http://pngu.mgh.harvard.edu/~purcell/plink/).

## Results

### The *STAT4* SNP rs7574865 modulates susceptibility to CD but not to UC

In all three subgroups (CD, UC, and controls), the allele frequencies of the *STAT4* SNPs rs11889341, rs7574865, rs7568275, rs8179673, rs10181656, rs7582694 and rs10174238 were in accordance with the predicted Hardy-Weinberg equilibrium (Table 4). Overall, we observed no significant differences in the frequencies of seven SNPs investigated in UC patients compared to healthy controls (Table 4). In patients with CD, the analysis revealed a significant association of the *STAT4* SNP rs7574865 with decreased susceptibility to CD (p = 0.047, OR 0.86 [95% CI 0.74–0.99]) (Table 4); however, this significance would have been lost after Bonferroni correction, which has not been applied since all seven *STAT4* SNP were in very strong linkage disequilibrium in all three subgroups of the study population (Supplemental Tables S1, S2, S3) as also described before [27]. Moreover, for two other variants there was a trend towards association with decreased CD susceptibility (rs7568275, p = 0.058, OR 0.86 [95% CI 0.74–1.00]; rs10174238, p = 0.057, OR 0.86 [95% CI 0.75–1.00]). For all other *STAT4* SNPs investigated, we could not demonstrate significant differences comparing the allele frequencies of CD patients with those of healthy controls (Table 4). Moreover, analysis of haplotypes consisting of SNPs within the *STAT4* gene did not detect any significant differences between CD and UC, respectively, when compared to the control group (Supplemental Tables S4 and S5).

**Table 4:** Associations of *STAT4* gene markers in CD and UC case-control association studies.

SNP	Minor allele	Crohn's diseasen = 857			Ulcerative colitisn = 464			Controlsn = 1383
		MAF	p value	OR [95% CI]	MAF	p value	OR [95% CI]	MAF
rs11889341	T	0.194	0.11	0.88 [0.76–1.03]	0.215	0.96	1.01 [0.84–1.20]	0.214
rs7574865	T	0.190	**0.047**	0.86 [0.74–0.99]	0.214	1.00	1.00 [0.83–1.20]	0.215
rs7568275	G	0.193	0.058	0.86 [0.74–1.00]	0.212	0.78	0.97 [0.81–1.17]	0.217
rs8179673	C	0.198	0.12	0.89 [0.76–1.03]	0.215	0.93	0.99 [0.82–1.18]	0.217
rs10181656	G	0.197	0.12	0.89 [0.76–1.03]	0.211	0.74	0.96 [0.80–1.16]	0.217
rs7582694	C	0.197	0.15	0.89 [0.77–1.04]	0.212	0.89	0.98 [0.82–1.18]	0.215
rs10174238	G	0.202	0.057	0.87 [0.75–1.00]	0.229	0.85	1.02 [0.85–1.22]	0.226

Minor allele frequencies (MAF), allelic test *P*-values, and odds ratios (OR, shown for the minor allele) with 95% confidence intervals (CI) are depicted for both the CD and UC case-control cohorts.

### Associations between *STAT4* genotype status and the CD phenotype

Based on the significant association of the *STAT4* SNP rs7574865 with the susceptibility to CD, we next performed a detailed genotype-phenotype analysis in a subgroup of n = 622 phenotypically well-characterized CD patients carrying SNP rs7574865. CD patients homozygous for rs7574865 showed a significant younger age at disease onset (23.2 years ±7.5) compared to wildtype patients (27.9 years ±12.3; p = 0.021) and also heterozygous patients (29.0 years ±12.4; p = 0.007). In addition, we observed significantly more male patients in the subcohort of homozygous carriers of the SNP rs7574865 compared to the wildtype patients (p = 0.040) and heterozygous patients (p = 0.005; Table 5). CD patients homozygous for rs7574865 were found to have significantly more frequent colonic disease compared to wildtype patients (p = 0.008) and heterozygous carriers (p = 0.033; Table 6). Moreover, none of the homozygous carriers demonstrated isolated CD of the terminal ileum. In contrast, the analysis revealed no significant associations with other phenotypic disease characteristics such as body mass index (BMI), incidence of stenoses and fistulas, use of immunosuppressive agents, or extraintestinal manifestations (Tables 5 and 6).

**Table 5:** Association between the *STAT4* rs7574865 gene variant and demographic characteristics of the CD cohort.

rs7574865Crohn's disease	(1)GG(n = 399)	(2)GT(n = 203)	(3)TT(n = 20)	(1) vs. (2)p valueOR	(1) vs. (3)p valueOR	(2) vs. (3)p valueOR	(1) vs. (2+3)p valueOR
**Male sex**	203/399 (51.0%)	84/203 (41.0%)	15/20 (75.0%)	**0.031**	**0.040**	**0.005**	0.132
				0.68 CI (0.48–0.96)	2.90 CI (1.03–8.12)	4.25 (1.49–12.14)	0.77 (0.56–1.07)
**Age (yr)**							
Mean ± SD	40.7±13.3	42.0±12.9	34.4±7.2	0.225	**0.001**	**0.0002**	0.528
Range	16–82	18–82	21–49				
**Age at diagnosis (yr)**							
Mean ± SD	27.9±12.3	29.0±12.4	23.2±7.5	0.308	**0.021**	**0.007**	0.551
Range	6–71	11–78	13–37				
**Disease duration (yr)**							
Mean ± SD	12.7±9.1	13.1±8.4	10.8±4.3	0.643	0.103	0.067	0.823
Range	0–46	1–42	2–19				
**BMI**							
Mean ± SD	23.2±4.3	22.8±4.2	23.4±3.3	0.312	0.808	0.487	0.366
Range	13–41	16–34	16–29				

**Table 6:** Association between the *STAT4* rs7574865 gene variant and CD characteristics based on the Montreal classification ^40^.

rs7574865	(1)	(2)	(3)	(1) vs. (2)	(1) vs. (3)	(2) vs. (3)	(1) vs. (2+3)
	GG	GT	TT	p value	p value	p value	p value
Crohn's disease	(n = 399)	(n = 203)	(n = 20)	OR	OR	OR	OR
**Age at diagnosis**							
≤16 years (A1)	45/383 (11.7%)	21/193 (10.9%)	4/18 (22.2%)	0.8900.92 CI (0.53–1.59)	0.2562.15 CI (0.68–6.80)	0.2412.34 CI (0.70–7.77)	0.8960.95 CI (0.56–1.59)
17–40 years (A2)	286/383 (74.7%)	142/193 (73.6%)	14/18 (78.8%)	0.8400.94 CI (0.94–1.40)	1.000	1.000	0.4460.86 CI (0.59–1.25)
>40 years (A3)	52/383 (13.6%)	30/193 (15.5%)	0	0.2481.33 CI (0.82–2.18)	1.19 CI (0.38–3.69)0.146	1.26 CI (0.40–4.00)0.083	1.0000.99 CI (0.61–1.60)
**Location**							
Terminal ileum (L1)	45/385 (11.7%)	31/192 (16.1%)	0	0.1511.45 CI (0.89–2.38)	0.242	0.082	0.3051.31 CI (0.80–2.14)
Colon (L2)	37/385 (9.6%)	25/192 (13.0%)	6/18 (33.3%)	0.2531.41 CI (0.82–2.42)	**0.008**4.60 CI (1.63–12.96)	**0.033**3.32 CI (1.14–9.64)	0.0791.63 CI (0.98–2.71)
Ileocolon (L3)	230/385 (59.7%)	115/192 (59.9%)	8/18 (44.4%)	1.0001.01 CI (0.71–1.43)	0.2250.54 CI (0.21–1.40)	0.2200.54 CI (0.20–1.42)	0.7940.95 CI (0.68–1.34)
Upper GI (L4)	10/385 (2.6%)	0	0	**0.035**	1.000	-	-
Terminal ileum and Upper GI (L1+L4)	10/385(2.6%)	2/192 (1.0%)	0	0.3540.39 CI (0.09–1.82)	1.000	1.000	0.2300.36 CI (0.08–1.66)
Colon and Upper GI (L2+L4)	4/385 (1.0%)	3/192 (1.6%)	0	0.6911.51 CI (0.33–6.82)	1.000	1.000	0.7021.38 CI (0.31–6.23)
Ileocolon and Upper GI (L3+L4)	49/385 (12.7%)	16/192 (8.3%)	4/18 (22.2%)	0.1230.62 CI (0.34–1.13)	0.2751.96 CI (0.62–6.20)	0.0763.14 CI (0.92–10.68)	0.2840.72 CI (0.42–1.25)
**Behaviour** 1							
Non-stricturing, Non-penetrat (B1)	82/369 (22.2%)	45/184 (24.5%)	2/19 (10.5%)	0.5921.13 CI (0.75–1.72)	0.3890.41 CI (0.09–1.82)	0.2540.36 CI (0.08–1.64)	0.8351.05 CI (0.70–1.59)
with perianal f. (B1p)	8/369 (2.2%)	4/184 (2.2%)	0	1.0001.00 CI (0.30–3.37)	1.000	1.000	1.0000.91 CI (0.27–3.05)
Stricturing (B2)	100/369 (27.1%)	39/184 (21.2%)	6/19 (31.6%)	0.1460.72 CI (0.47–1.10)	0.7921.24 CI (0.46–3.35)	0.3821.72 CI (0.61–4.81)	0.2280.77 CI (0.51–1.15)
with perianal f. (B2p)	10/369 (2.7%)	9/184 (4.9%)	0	0.2171.85 CI (0.74–4.63)	1.000	1.000	0.3301.66 CI (0.67–4.17)
Penetrating (B3)	152/369 (41.2%)	81/184 (44.0%)	10/19 (52.6%)	0.5841.12 CI (0.78–1.60)	0.3481.59 CI (0.63–4.00)	0.4801.41 CI (0.55–3.64)	0.4271.16 CI (0.82–1.64)
with perianal f. (B3p)	17/369 (4.6%)	6/184 (3.3%)	1/19 (5.3%)	0.5080.70 CI (0.27–1.80)	0.6031.15 CI (0.14–9.13)	0.5031.65 CI (0.19–14.45)	0.6640.74 (0.30–1.81)
**Use of immunosuppressive agents**	307/376 (82.0%)	154/188 (82.0%)	16/20 (80.0%)	1.0001.01 CI (0.65–1.60)	0.7720.90 CI (0.29–2.77)	0.7670.88 CI (0.28–2.81)	1.0001.00 CI (0.65–1.56)
**Use of infliximab**	156/397 (39.0%)	80/199 (40.0%)	9/20 (45.0%)	0.8591.04 CI (0.73–1.47)	0.6441.26 CI (0.51–3.12)	0.8121.22 CI (0.48–3.07)	0.7961.06 CI (0.75–1.48)
**Fistula**	187/369 (50.7%)	102/184 (55.4%)	11/19 (57.9%)	0.3211.21 CI (0.85–1.73)	0.6411.34 CI (0.53–3.40)	1.0001.10 CI (0.42–2.87)	0.2571.22 CI (0.87–1.72)
**Stenosis**	241/364 (66.2%)	123/182 (67.6%)	12/19 (63.2%)	0.7731.06 CI (0.73–1.55)	0.8060.87 CI (0.34–2.28)	0.7980.82 CI (0.31–2.20)	0.8531.04 CI (0.72–1.50)
**Abscesses**	112/335 (33.0%)	71/169 (42.0%)	7/19 (37.0%)	0.0631.44 CI (0.99–2.11)	0.8051.16 CI (0.44–3.03)	0.8070.80 CI (0.30–2.14)	0.0721.41 CI (0.98–2.04)
**Surgery because of CD**	219/355 (62.0%)	116/177 (66.0%)	10/18 (56.0%)	0.3931.18 CI (0.81–1.71)	0.6260.78 CI (0.30–2.01)	0.4420.66 CI (0.25–1.75)	0.1571.34 CI (0.91–1.93)
**Positive family history of IBD**	55/291 (18.9%)	17/139 (12.2%)	1/13 (7.7%)	0.0980.60 CI (0.33–1.07)	0.4750.36 CI (0.05–2.81)	1.0000.60 CI (0.07–4.89)	0.0600.58 CI (0.32–1.02)

For each variable, the number of patients with complete information on this particular disease variable included in the analysis is given.

1 Disease behaviour was defined according to the Montreal classification ^40^. A stricturing disease phenotype was defined as presence of stenosis without penetrating disease. The diagnosis of stenosis was made surgically, endoscopically, or radiologically (using MR enteroclysis).

2 Immunosuppressive agents included azathioprine, 6-mercaptopurine, methotrexate, and/or infliximab.

3 Only surgery related to CD-specific problems (e.g. fistulectomy, colectomy, ileostomy) was included.

### Analysis for epistasis with *IL23R*

Given that IL-23, a cytokine involved in Th17 cell differentiation, activates not only *STAT3* but also to a lesser degree *STAT4*, we next investigated potential epistasis with the IBD susceptibility gene *IL23R*. Analysis for 10 SNPs in the *IL23R* region which have previously shown to significantly influence CD susceptibility [14] found weak interactions between the *STAT4* SNPs rs8179673, rs7582694 and rs10174238 with the coding *IL23R* SNP rs11209026 = p.Arg181Gln in CD (Supplemental Table S6) but significance was lost after correction for multiple comparisons. Interestingly, similar to the effect of the *STAT4* SNP rs7574865 shown in this study, the *IL23R* SNP rs11209026 is protective for CD susceptibility. However, none of the three *STAT4* SNPs with epistasis to the *IL23R* SNP rs11209026 were independently associated with CD, although there was a trend for association of CD with rs10174238 (rs10174238: p = 0.057; rs8179673: p = 0.12; rs7582694: p = 0.15; Table 4). Furthermore, no significant interaction was observed between *STAT4* and *IL23R* SNPs in UC (Supplemental Table S7).

## Discussion

Here, we present the first detailed genotype-phenotype analysis of *STAT4* gene variants in a large Caucasian IBD cohort. In line with previous reports of significant associations of genetic variants in the *STAT4* gene with the risk for autoimmune diseases such as SLE, rheumatoid arthritis [23], [27], [32] and psoriasis, [23], [36] our study could identify the *STAT4* SNP rs7574865 also to be associated with the susceptibility to CD. However, this association to CD did not reach the extent of significance or clinical relevance shown for other CD susceptibility genes such as *NOD2*/*CARD15* and *IL23R* [5], [13], [14], [41]–[43]. For all other *STAT4* gene variants investigated (rs11889341, rs7568275, rs8179673, rs10181656, rs7582694 and rs10174238), our analysis did not reveal any significant association with CD or UC susceptibility. However, there was a trend towards an association in the case of rs7568275 and rs10174238 in CD, which can be explained by the strong linkage disequilibrium between all seven *STAT4* SNPs [27] investigated in our study. A smaller Spanish study including 700 IBD patients also reported a significant association of the *STAT4* variant rs7574865 with CD susceptibility, although rs7574865 was in this study a risk allele and not protective [38]. In contrast, a very recent Korean study found no association of *STAT4* SNPs with CD but a weak association of the *STAT4* SNP rs925847 with susceptibility to UC (P = 0.025; OR = 0.63). [44] Similar to the findings in CD in our study, *STAT4* was protective against UC in the Korean study. [44] In contrast, another very recent study from Spain found no association of the *STAT4* SNP rs7574865 with CD and UC [45], while an extended meta-analysis showed a disease association of this SNP with UC and not CD in the Spanish population. [45] Considering the limited data of *STAT4* gene variants in IBD patients and the conflicting results from the Spanish studies, the impact of *STAT4* on IBD susceptibility seems to be much more limited than that of *STAT3* variants.

In addition, this study demonstrated weak epistasis between the CD-protective *IL23R* variant rs11209026 with several *STAT4* SNPs (Supplementary Table S6). However, none of these gene-gene interactions remained significant after Bonferroni correction. Interestingly, similar to our findings, opposing effects of *STAT3* variants on different Th17-mediated autoimmune diseases have been reported. A very recent genome-wide association study identified *STAT3* as a new susceptibility gene for multiple sclerosis (MS), a disorder in which the Th17 pathway plays a major role. [46] Remarkably, in MS, the G allele of the *STAT3* SNP rs744166 is the risk allele, whereas in CD, the A allele has been found to increase disease susceptibility. [47] Thus, it is possible that a SNP can confer opposite effects in different disorders within the same pathway. Further genotype-phenotype studies in large patient cohorts of different ethnic origin will therefore be required before final conclusion on the influence of *STAT4* on IBD susceptibility can be drawn.

In our study, CD patients homozygous for the SNP rs7574865 were significantly younger at disease onset compared to wildtype and heterozygous patients. Moreover, CD patients homozygous for rs7574865 were found to have significantly more frequent colonic disease compared to wildtype patients (p = 0.008) and heterozygous carriers (p = 0.033). It is therefore of special interest that particularly the *STAT4* SNP rs7574865, which has previously shown to be the most significant SNP in the association studies for other IBD-associated autoimmune diseases such as SLE, rheumatoid arthritis [23], [27], [32] and Sjögren's disease, [23], [34] is associated with both CD susceptibility and CD phenotype. However, similar to *STAT4*, other IBD susceptibility genes such as *IL23R*, *IL2/IL21*, *STAT3*, and *PTPN2* are associated with other autoimmune diseases. [48]–[53]

Regarding the disease-modifying effect of genetic variants in the *STAT4* region on IBD observed in our study, one might hypothesize whether the *STAT4* risk allele has different expression levels or functional effects in different effector cells. A recent study demonstrated a significant association of the *STAT4* risk allele with overexpression of *STAT4* in primary cells of mesenchymal origin such as osteoblasts but not in B cells. [54] This might indicate the potential presence of a tissue-specific intragenic enhancer and cell-type-specific effects of different *STAT4* gene variants on STAT4 expression levels. This would be in line with previous studies demonstrating that STAT4 isoforms differentially regulate Th1 cytokine production with STAT4β promoting greater colonic inflammation and tissue destruction which correlates with STAT4 isoform-dependent expression of TNF-α and GM-CSF *in vitro* and *in vivo*, but not Th1 expression of IFN-γ or Th17 expression of IL-17. [30] Further investigations on the effect of genetic variants on the expression levels and splicing isoforms in both T cells and B cells as well as intestinal epithelial cells will therefore be necessary. Moreover, based on previous studies [55] reporting an increased sensitivity to IFN-α in lupus patients carrying the risk variant of *STAT4*, one might also speculate whether this mechanism might contribute to increased mucosal inflammation in IBD patients and to the response of immunosuppressive and immunomodulatory therapies.

In summary, our results identified the *STAT4* SNP rs7574865 as a disease-modifying gene variant in CD. Homozygous carriers of the minor allele of rs7574865 have an earlier CD onset and more often colonic disease than carriers of the major allele. Further studies on expression and regulation of STAT4 in the intestinal mucosa will be required to investigate the functional consequences of *STAT4* gene variants in more detail.

## Supporting Information

Table S1 Linkage disequilibrium (LD) between *STAT4* SNPs in controls. Values are given as D′/r^2^.
(0.12 MB DOC)

Table S2 LD between *STAT4* SNPs in CD patients. Values are given as D′/r^2^.
(0.12 MB DOC)

Table S3 LD between *STAT4* SNPs in UC patients. Values are given as D′/r^2^.
(0.12 MB DOC)

Table S4 Haplotype analysis for *STAT4* SNPs in the CD case-control cohort.
(0.12 MB DOC)

Table S5 Haplotype analysis for *STAT4* SNPs in the UC case-control cohort.
(0.12 MB DOC)

Table S6 Epistasis between *STAT4* and *IL23R* SNPs in the CD case-control cohort.
(0.17 MB DOC)

Table S7 Epistasis between *STAT4* and *IL23R* SNPs in the UC case-control cohort.
(0.16 MB DOC)
