# Supplementary material for: Evidence for STAT4 as a Common Autoimmune Gene: rs7574865 Is Associated with Colonic Crohn's Disease and Early Disease Onset
Source: PLoS One. 2010 Apr 29;5(4):e10373. doi: 10.1371/journal.pone.0010373 (PMC2861592; doi:10.1371/journal.pone.0010373)
Supplement: Table S1 — Linkage disequilibrium (LD) between STAT4 SNPs in controls. Values are given as D′/r2. (0.12 MB DOC) [file pone.0010373.s001.doc]

**Supporting information**

**Supplemental Table S1.** Linkage disequilibrium (LD) between *STAT4* SNPs in controls. Values are given as D'/r².

|  | **rs11889341** | **rs7574865** | **rs7568275** | **rs8179673** | **rs10181656** | **rs7582694** | **rs10174238** |
| --- | --- | --- | --- | --- | --- | --- | --- |
| **rs11889341** | * |  |  |  |  |  |  |
| **rs7574865** | 0.99/0.97 | * |  |  |  |  |  |
| **rs7568275** | 0.99/0.97 | 1.00/0.99 | * |  |  |  |  |
| **rs8179673** | 0.99/0.96 | 1.00/0.98 | 0.99/0.98 | * |  |  |  |
| **rs10181656** | 0.99/0.96 | 1.00/0.99 | 0.99/0.99 | 0.99/0.98 | * |  |  |
| **rs7582694** | 0.98/0.96 | 1.00/0.99 | 1.00/0.98 | 1.00/0.98 | 1.00/0.98 | * |  |
| **rs10174238** | 0.96/0.86 | 0.97/0.88 | 0.96/0.88 | 0.96/0.89 | 0.96/0.88 | 0.97/0.88 | * |
