# Supplementary material for: Evidence for STAT4 as a Common Autoimmune Gene: rs7574865 Is Associated with Colonic Crohn's Disease and Early Disease Onset
Source: PLoS One. 2010 Apr 29;5(4):e10373. doi: 10.1371/journal.pone.0010373 (PMC2861592; doi:10.1371/journal.pone.0010373)
Supplement: Table S3 — LD between STAT4 SNPs in UC patients. Values are given as D′/r2. (0.12 MB DOC) [file pone.0010373.s003.doc]

**Supplemental Table S3.** LD between *STAT4* SNPs in UC patients. Values are given as D'/r².

|  | **rs11889341** | **rs7574865** | **rs7568275** | **rs8179673** | **rs10181656** | **rs7582694** | **rs10174238** |
| --- | --- | --- | --- | --- | --- | --- | --- |
| **rs11889341** | * |  |  |  |  |  |  |
| **rs7574865** | 0.98/0.96 | * |  |  |  |  |  |
| **rs7568275** | 0.98/0.96 | 1.00/1.00 | * |  |  |  |  |
| **rs8179673** | 0.98/0.96 | 1.00/1.00 | 1.00/1.00 | * |  |  |  |
| **rs10181656** | 0.98/0.96 | 1.00/1.00 | 1.00/1.00 | 1.00/1.00 | * |  |  |
| **rs7582694** | 0.98/0.96 | 1.00/1.00 | 1.00/1.00 | 1.00/1.00 | 1.00/1.00 | * |  |
| **rs10174238** | 0.95/0.82 | 0.95/0.85 | 0.95/0.84 | 0.95/0.84 | 0.95/0.84 | 0.95/0.84 | * |
