# Supplementary material for: Evidence for STAT4 as a Common Autoimmune Gene: rs7574865 Is Associated with Colonic Crohn's Disease and Early Disease Onset
Source: PLoS One. 2010 Apr 29;5(4):e10373. doi: 10.1371/journal.pone.0010373 (PMC2861592; doi:10.1371/journal.pone.0010373)
Supplement: Table S4 — Haplotype analysis for STAT4 SNPs in the CD case-control cohort. (0.12 MB DOC) [file pone.0010373.s004.doc]

**Supplemental Table S4.** Haplotype analysis for *STAT4* SNPs in the CD case-control cohort

| **Haplotype combination** | **Omnibus p-value** |
| --- | --- |
| rs11889341-rs7574865 | 0.22 |
| rs7574865-rs7568275 | 0.12 |
| rs7568275-rs8179673 | 0.17 |
| rs8179673-rs10181656 | 0.22 |
| rs10181656-rs7582694 | 0.25 |
| rs7582694-rs10174238 | 0.45 |
| rs11889341-rs7574865-rs7568275 | 0.19 |
| rs7574865-rs7568275-rs8179673 | 0.21 |
| rs7568275-rs8179673-rs10181656 | 0.19 |
| rs8179673-rs10181656-rs7582694 | 0.23 |
| rs10181656-rs7582694-rs10174238 | 0.45 |
| rs11889341-rs7574865-rs7568275-rs8179673 | 0.26 |
| rs7574865-rs7568275-rs8179673-rs10181656 | 0.20 |
| rs7568275-rs8179673-rs10181656-rs7582694 | 0.20 |
| rs10181656-rs7582694-rs10174238-rs10174238 | 0.44 |
| rs11889341-rs7574865-rs7568275-rs8179673-rs10181656 | 0.27 |
| rs7574865-rs7568275-rs8179673-rs10181656-rs7582694 | 0.22 |
| rs7568275-rs8179673-rs10181656-rs7582694-rs10174238 | 0.43 |
| rs11889341-rs7574865-rs7568275-rs8179673-rs10181656-rs7582694 | 0.27 |
| rs7574865-rs7568275-rs8179673-rs10181656-rs7582694-rs10174238 | 0.46 |
| rs11889341-rs7574865-rs7568275-rs8179673-rs10181656-rs7582694-rs10174238 | 0.41 |
