# Supplementary material for: Evidence for STAT4 as a Common Autoimmune Gene: rs7574865 Is Associated with Colonic Crohn's Disease and Early Disease Onset
Source: PLoS One. 2010 Apr 29;5(4):e10373. doi: 10.1371/journal.pone.0010373 (PMC2861592; doi:10.1371/journal.pone.0010373)
Supplement: Table S5 — Haplotype analysis for STAT4 SNPs in the UC case-control cohort. (0.12 MB DOC) [file pone.0010373.s005.doc]

**Supplemental Table S5.** Haplotype analysis for *STAT4* SNPs in the UC case-control cohort

| **Haplotype combination** | **Omnibus p-value** |
| --- | --- |
| rs11889341-rs7574865 | 0.49 |
| rs7574865-rs7568275 | 0.37 |
| rs7568275-rs8179673 | 0.32 |
| rs8179673-rs10181656 | 0.34 |
| rs10181656-rs7582694 | 0.35 |
| rs7582694-rs10174238 | 0.60 |
| rs11889341-rs7574865-rs7568275 | 0.44 |
| rs7574865-rs7568275-rs8179673 | 0.31 |
| rs7568275-rs8179673-rs10181656 | 0.30 |
| rs8179673-rs10181656-rs7582694 | 0.33 |
| rs10181656-rs7582694-rs10174238 | 0.47 |
| rs11889341-rs7574865-rs7568275-rs8179673 | 0.36 |
| rs7574865-rs7568275-rs8179673-rs10181656 | 0.31 |
| rs7568275-rs8179673-rs10181656-rs7582694 | 0.31 |
| rs10181656-rs7582694-rs10174238-rs10174238 | 0.37 |
| rs11889341-rs7574865-rs7568275-rs8179673-rs10181656 | 0.36 |
| rs7574865-rs7568275-rs8179673-rs10181656-rs7582694 | 0.31 |
| rs7568275-rs8179673-rs10181656-rs7582694-rs10174238 | 0.34 |
| rs11889341-rs7574865-rs7568275-rs8179673-rs10181656-rs7582694 | 0.37 |
| rs7574865-rs7568275-rs8179673-rs10181656-rs7582694-rs10174238 | 0.34 |
| rs11889341-rs7574865-rs7568275-rs8179673-rs10181656-rs7582694-rs10174238 | 0.51 |
