# Supplementary material for: Evidence for STAT4 as a Common Autoimmune Gene: rs7574865 Is Associated with Colonic Crohn's Disease and Early Disease Onset
Source: PLoS One. 2010 Apr 29;5(4):e10373. doi: 10.1371/journal.pone.0010373 (PMC2861592; doi:10.1371/journal.pone.0010373)
Supplement: Table S6 — Epistasis between STAT4 and IL23R SNPs in the CD case-control cohort. (0.17 MB DOC) [file pone.0010373.s006.doc]

**Supplemental Table S6.** Epistasis between *STAT4* and *IL23R* SNPs in the CD case-control cohort

| ***STAT4*-SNP** | ***IL23R*-**  **rs1004819** | ***IL23R*-**  **rs7517847** | ***IL23R*-**  **rs10489629** | ***IL23R*-**  **rs2201841** | ***IL23R*-**  **rs11465804** | ***IL23R*-**  **rs11209026** | ***IL23R*-**  **rs1343151** | ***IL23R*-**  **rs10889677** | ***IL23R*-**  **rs11209032** | ***IL23R*-**  **rs1495965** |
| --- | --- | --- | --- | --- | --- | --- | --- | --- | --- | --- |
| **rs11889341** | 0.10 | 0.96 | 0.73 | 0.60 | 0.17 | *0.08* | 0.74 | 0.70 | 0.71 | 0.53 |
| **rs7574865** | 0.92 | 0.95 | 0.78 | 0.74 | 0.22 | 0.10 | 0.48 | 0.87 | 0.60 | 0.69 |
| **rs7568275** | 0.84 | 0.79 | 0.64 | 0.56 | 0.30 | 0.14 | 0.50 | 0.74 | 0.84 | 0.53 |
| **rs8179673** | 0.99 | 0.82 | 0.74 | 0.86 | 0.11 | **0.04** | 0.57 | 0.96 | 0.60 | 0.82 |
| **rs10181656** | 0.90 | 0.80 | 0.71 | 0.76 | 0.12 | *0.05* | 0.63 | 0.94 | 0.70 | 0.78 |
| **rs7582694** | 0.83 | 0.91 | 0.78 | 0.71 | 0.10 | **0.04** | 0.77 | 0.90 | 0.76 | 0.65 |
| **rs10174238** | 0.89 | 0.90 | 0.67 | 0.95 | 0.13 | **0.04** | 0.75 | 0.80 | 0.57 | 0.55 |

Significant p values <0.05 are depicted in bold, suggestive p values < 0.10 are depicted in Italic font. None of the associations remained significant after Bonferroni correction.
