# Supplementary material for: Evidence for STAT4 as a Common Autoimmune Gene: rs7574865 Is Associated with Colonic Crohn's Disease and Early Disease Onset
Source: PLoS One. 2010 Apr 29;5(4):e10373. doi: 10.1371/journal.pone.0010373 (PMC2861592; doi:10.1371/journal.pone.0010373)
Supplement: Table S7 — Epistasis between STAT4 and IL23R SNPs in the UC case-control cohort. (0.16 MB DOC) [file pone.0010373.s007.doc]

**Supplemental Table S7.** Epistasis between *STAT4* and *IL23R* SNPs in the UC case-control cohort

| ***STAT4*-SNP** | ***IL23R*-**  **rs1004819** | ***IL23R*-**  **rs7517847** | ***IL23R*-**  **rs10489629** | ***IL23R*-**  **rs2201841** | ***IL23R*-**  **rs11465804** | ***IL23R*-**  **rs11209026** | ***IL23R*-**  **rs1343151** | ***IL23R*-**  **rs10889677** | ***IL23R*-**  **rs11209032** | ***IL23R*-**  **rs1495965** |
| --- | --- | --- | --- | --- | --- | --- | --- | --- | --- | --- |
| **rs11889341** | 0.95 | 0.79 | 0.73 | 0.45 | 0.49 | 0.21 | 0.61 | 0.37 | 0.72 | 0.46 |
| **rs7574865** | 0.89 | 0.82 | 0.69 | 0.49 | 0.55 | 0.24 | 0.53 | 0.40 | 0.70 | 0.41 |
| **rs7568275** | 0.86 | 0.82 | 0.68 | 0.52 | 0.58 | 0.25 | 0.54 | 0.42 | 0.66 | 0.42 |
| **rs8179673** | 0.90 | 0.72 | 0.60 | 0.50 | 0.57 | 0.25 | 0.50 | 0.41 | 0.77 | 0.41 |
| **rs10181656** | 0.89 | 0.77 | 0.64 | 0.49 | 0.58 | 0.26 | 0.48 | 0.41 | 0.73 | 0.36 |
| **rs7582694** | 0.81 | 0.77 | 0.62 | 0.56 | 0.55 | 0.24 | 0.52 | 0.46 | 0.66 | 0.42 |
| **rs10174238** | 0.74 | 0.82 | 0.77 | 0.23 | 0.82 | 0.26 | 0.66 | 0.17 | 0.94 | 0.30 |
